# Listen to the heart or mind first? Examining sequential coping mechanisms among Indians during the COVID-19 pandemic

**DOI:** 10.3389/fpsyg.2023.1104973

**Published:** 2023-03-01

**Authors:** Sanchita Srivastava, Puja Upadhaya, Renuka Jain

**Affiliations:** ^1^CHRIST (Deemed to be University), Delhi-NCR, Ghaziabad, India; ^2^Independent Researcher, New Delhi, India

**Keywords:** stress, coping, wellbeing, COVID-19, pandemic, lockdown, India

## Abstract

The present study examines the mediating role of emotion-focused and problem-focused coping between stress and psychological well-being during the COVID-19 pandemic. The sample comprised 501 (312 women and 184 men aged between 18 and 42) Indians who experienced the first-ever continued lockdown in India during the COVID-19 pandemic. The results of this study confirmed the presence of perceived stress due to the lockdown and pandemic among participants. Furthermore, perceived stress, coping including emotion-focused and problem-focused, and psychological well-being were found to be interrelated. The serial mediation analysis revealed that participants dealt with stress by choosing emotion-focused coping first as an immediate resort. After a reappraisal of stress-inducing situations, they used problem-focused coping, and this sequence of constant coping mechanisms helped maintain their psychological well-being. The findings of this study can be applied to develop strategies for people’s mental health by public health organizations and health professionals.

## Introduction

In December 2019, an outbreak of novel coronavirus in Wuhan, China, was soon declared a pandemic by WHO (World Health Organization, 2020). India was affected by this disease, like many countries, after detecting its first case in March 2020. The Indian government responded swiftly to battle with the novel coronavirus. Appropriate measures were taken following the guidelines of WHO and Indian Council of Medical Research (ICMR), and India observed its first 1-day lockdown, named “Janta Curfew” on 22nd March 2020 (Chandna and Basu, 2020). Later, the Government of India officially announced its first phase of nationwide lockdown from 25th March 2020 to 14th April 2020 (Gettlement and Schults, 2020), which continued until 31st May 2020 (Lancet, 2020). This continued lockdown period had strict guidelines for staying at home, with restricted communication operations for essential employees. The closure of schools, colleges, shops, businesses, markets, and offices caused distress to many Indians.

These sudden life changes pose two major challenges for all Indians: first, to protect oneself and family members from this novel infectious disease with little information about its severity, mortality chances, and no available treatment/vaccine. Second, to suddenly stay at home without proper planning and preparation during lockdown phases. This lockdown has put many lives at a halt in canceling examinations (Nagari, 2020), facing a financial crisis (ET 2020), and fearing uncertainty about the future in terms of jobs, health, and other life events. These challenges can be considered extraordinary for them and may have implications for their physical and mental health. Studies on individuals identified as potential carriers of the virus and who were also quarantined during the epidemics and pandemics confirm the negative impact of social isolation on quarantined individuals’ mental health and well-being (Banerjee, 2020; Brooks and Geyer, 2020; Racine et al., 2022).

The history of pandemic diseases speaks volumes about being responsible for the severity and destruction caused to humans worldwide. Panic, stress, anxiety, and fear of losing lives are common yet profound responses manifested in pandemic-like situations (Grover et al., 2020; Roy et al., 2020). The unexpected lockdown across nations, including India, also has repercussions on individuals’ psychological and social well-being (Grover et al., 2020; Verma and Mishra, 2020; Rehman et al., 2021). Advisories from many top public and welfare organizations clearly stated the pertinent mental health issues and ways to combat them and protect physical health by following the ‘new normal’^1^. However, the impact of the continued lockdown situation on an individual’s stress perception and well-being with its unique complexities is still in its nascent stage.

Lazarus and Folkman (1984, p. 19) conceptualized stress as a mediation between the person and environment and differentiated between physiological and psychological stress. Psychological stress is “a particular relationship between the person and the environment that the person appraises as taxing or exceeding their resources and endangering their well-being.” An individual can appraise an event as a threat or challenge. The threat can lead to negative emotions such as fear and anxiety, which may inflict avoidance. However, the challenge can lead to positive emotions such as hope and confidence, which may motivate the individual to face the stressor depending on the perceived danger and available resources to deal with it. Some events can be appraised as threats and challenges, as they evoke mixed emotions.

Similarly, a lockdown and pandemic could be conceptualized as a complex stressor or event that could be perceived as either a threat or challenge and threat-challenge depending on various intervening factors. The present study examined how COVID-19 pandemic-related lockdown duration is perceived as stressful among Indians. Prolonged episodes of stress could be detrimental to individual’s psychological well-being. Selective coping strategies are required to deal with stress perceptions, as individual differences were found in the severity of consequences experienced by people making some groups more vulnerable than others (Cohen and Janicki-Deverts, 2012).

Lazarus and Folkman (1984, p.141) conceptualized coping as a process. They defined coping as “constantly changing cognitive and behavioral efforts to manage external and/or internal demands that are appraised as taxing or exceeding the person’s resources.” They identified coping as a dynamic process rather than a static response. They stressed an individual’s “changing” efforts to deal with the stressor or stressful situation progress with time. Coping literature identifies various ways of coping utilized by individuals to deal effectively with stressors. Researchers have been categorizing these coping styles as emotion-focused, and problem-focused coping, where the former is concerned with managing instinctive emotions and reactions due to the encounter of the stressful event, and the latter intends to find an option to solving the stressor (Carver and Scheier, 1994; Baker and Berenbaum, 2007; Herman and Tetrick, 2009; Schoenmakers et al., 2015; Li, 2020). Problem-focused coping encompasses active coping, planning, and instrumental support. Effective coping styles can successfully reduce stress perceptions of individuals (Lazarus and Folkman, 1984). This effectiveness is also subjected to the careful selection of coping strategy as the nature of the stressor determines the choice of coping strategy. For example, in one situation choosing an emotion-focused coping might work well to overcome the initial cognitive burden, but sometimes if the resources are enough to intervene with the stressor directly, then problem-focused coping can be most effective (Ghane et al., 2016). Studies suggested that problem-focused coping is more effective than emotion-focused coping, so if the individual is engaged actively to overcome or resolve the source of the stressor, then it will lead to a positive outcome, on the contrary if the individual uses emotion-focused coping to avoid or reduce the overwhelming emotions then it is a temporary solution, but eventually, it will become ineffective (Penley et al., 2002). However, the nature of the stressor and its duration also play an important role in making the chosen coping strategy effective. For example, studies on grief and terminal illness show that emotion-focused coping is useful and sometimes the only way of coping applicable (Carr, 2020; Siegel et al., 2001).

Furthermore, these studies also suggested that choosing a mix of coping such as emotion-focused and problem-focused together, can make the coping meaningful and positive for its user. For example, the death of a loved one, getting the news of a life-threatening disease or terminal illness can make the individual overwhelmed and requires emotion-focused coping, such as seeking emotional support, positive reframing, or religion (Agbaria and Abu-Mokh, 2022; Butler et al., 2005; Carr and Mooney, 2021; Janson and Rohleder, 2017). After dealing with emotional reactions, the individual can look for the active problem-solving, planning, and gaining instrumental support (Shimazu and Schaufeli, 2007). On the contrary, in instances such as during COVID-19 outburst, people were recommended to maintain social distancing and avoid direct contact with a COVID patient. This simple step of not meeting a family member reduces the chances of contracting with COVID-19; using phones or virtual mediums to connect with others can be seen as active problem-solving. But as a result, the individual might feel loneliness, distress, and anxiousness for not physically meeting and taking care of the patient, which can be dealt with using emotion-focused coping such as venting or religion. Therefore, it is not a mandatory choice between problem-focused and emotion-focused. A combination of both is often effective and viable for dealing with stressors and protecting well-being (Lazarus, 1993; Cohen et al., 1995). The effectiveness of coping depends on the stressor’s nature, duration, situational factors, and individual personality. Therefore, it is apt to say that coping is situation/context-specific, and its effectiveness depends on multiple factors, not just the category of coping itself (Cohen et al., 1995; Shimazu and Schaufeli, 2007). Therefore, coping and stress share a significant and complex relationship as the perception of stressors is needed to activate the coping process.

Coping is context-specific or situation-specific, as the stressor’s nature, duration, personality, and existing resources will determine what action to take (Cohen et al., 1995). Witnessing a pandemic in the form of the COVID-19 virus was a stressor for the world, and an individual experiencing the lockdown, the contagious nature of the virus, and no existing cure had made it challenging for most people in the world. Coping is generally perceived as a fight-flight response to the stressor. However, coping is not a simple mechanism but a complex multidimensional construct that includes a primary and secondary appraisal of the event and constant reappraising until it gets over completely (Cohen et al., 1995). The individual does not necessarily stick to one coping strategy to deal with the stressor instead combines a variety of coping strategies from time to time with the feedback of the reappraisal of the stressor (Carver and Scheier, 1994; Cohen et al., 1995; Skinner et al., 2003; Li, 2020). Chen and Miller (2012) provided a significant perspective on coping among disadvantaged sections of society. They examined that with the help of “shift-persist” strategies use how a low-income group protects their health and well-being from constant adversities in life. This “shift-persist” coping suggests that when an individual cannot directly respond to or resolve the issues identified as stressors by the individual, then s/he first shifts their emotional responses from it by reframing the stressor or acknowledging and accepting it. Then constantly actively looking for support and solutions to the stressor to reduce or completely resolve it by developing flexibility and maintaining a positive perspective. This combination of constantly shifting their emotion and attention and then actively working toward it help them to deal with adversity with resilience and positively impacts their physical and psychological well-being (Chen, 2012). Carver and Scheier (1994, p.184) also mentioned about the coping strategies such as “emotion-focused coping can facilitate problem-focused coping by removing some of the distress that can hamper problem-focused efforts.” Ben-Zur (2020, p.1345) suggested that “emotion-focused coping may contribute to better functioning and long term health and satisfaction if it eventually helps to initiate problem-focused actions.”

The present study hypothesized that individuals would appraise the stressful situation and use various coping mechanisms to deal with it, and the types of coping will determine their psychological well-being. It hypothesized that individuals would first engage in various emotion-focused coping which will act as a buffer to deal with emotions and provide time to organize resources to deal with COVID-19 related stressors through problem-focused coping strategies. Through the path of choosing emotion-based coping and then indulging in problem-focused coping, individuals will be able to manage the stress that will eventually impact or determine their psychological well-being.

## Methods

### Participants and procedure

Demographic information is summarized in Table 1. A total of 501 participants contributed to this study. All participants were 18 years or above. Among the sample 312 (62.3%) were women, and 345 (68.8%) participants were between 18 and 42 years of age. Most of the participants were married 369 (71.9%).

**Table 1:** Demographic characteristics of participants (*n* = 501).

Variables	*n* (%)
**Gender**	
Male	189 (37.7%)
Female	312 (62.3%)
**Age**	
Young adults (18–35)	211 (42.12%)
Middle-aged (36–55)	244 (48.70%)
Older adults (56 and above)	46 (9.18)
**Marital status**	
Married	360 (71.9%)
Unmarried	136 (27.1%)
Other	5 (1%)
**Employment status**	
Employed	326 (65.1%)
Unemployed	11 (2.2%)
Homemaker	69 (37.8%)
Student	78 (15.6%)
Retired	17 (3.3%)
**Socioeconomic status**	
Upper	5 (1%)
Upper middle	200 (39.9%)
Middle	264 (52.7%)
Lower middle	30 (6%)
Lower	2 (0.4%)

Regarding employment status, around 65% of participants were employed, while 37.8% were homemakers and 15.6% were students. Most of the participants belong to the upper middle 200 (39.9%) and middle 264 (52.7%) socioeconomic status (SES) based on their self-reported categorization of the SES. Data were collected using convenience sampling distributed using social media platforms and WhatsApp groups. Therefore, the potential participants were limited to social media and WhatsApp users and the ability to understand survey questions in the English language. The survey was designed to collect online responses from the participants to maintain confidentiality and anonymity. The objective of the study and participation criteria (e.g., Indian nationals who were residing in India, 18 years and above age, experienced lockdown phases in India, etc.) were clearly stated on the survey’s introductory page. It mentioned anonymity, confidentiality, and the option to withdraw from participation at any time. Informed consent was taken before participation in the survey designed for all Indians who resided in India during the COVID-19 pandemic lockdown. This study received ethical approval from the Institutional Review Board.

### Measures

#### Perceived stress scale

Perceived stress scale (PSS-10) measures the stress perceptions of the participants. It is the 10-item measure considered best among the three versions of the PSS-10 (Cohen and Williamson, 1988). Responses are measured on a five-point Likert scale ranging from 0 (never) to 4 (very often). In this study, PSS-10 reliability has been noticed as 0.81, while the reliability of the original scale was 0.78 (Cohen and Williamson, 1988).

#### Brief COPE scale

A brief coping inventory was chosen for assessing 14 coping strategies of participants (Carver, 1997). Each coping strategy has two items. This brief measure is a short version of the coping measure developed by Carver et al. (1989), which is an effective tool for measuring health-related outcomes. These coping strategies are divided into two types of coping styles: problem-focused and emotion-focused (Li, 2020). The reliability of the original scale varies from 0.50 to 0.90 for all dimensions (Carver, 1997). The present study has noticed the reliability of 0.71 and 0.77 for problem-focused, and emotion-focused coping, respectively.

#### Psychological well-being scale

This scale has six dimensions: self-acceptance, positive relationship with others, autonomy, environmental mastery, purpose in life, and personal growth (Ryff, 1989). Each dimension has three items, and these items were scored on a five-point Likert scale ranging from 1 (strongly agree) to 5 (strongly disagree), where higher scores indicate a higher level of well-being. In this study, the reliability of the scale has been found as 0.75. Ryff (1989) mentioned the reliability of their scale low to modest, which varies from 0.33 to 0.56.

### Analysis

The relationship among perceived stress, psychological well-being, emotion-focused, and problem-focused coping was examined using PROCESS tool version 4.0 of Hayes (2021). The serial mediation model 6 was used to examine the relationship. In the analysis, 5,000 bootstraps samples with 95% of confidence intervals were used.

## Results

Descriptive statistics are presented in Table 1. The correlational analysis shows that perceived stress and psychological well-being share a negative relationship. On the other hand, psychological well-being is negatively correlated with emotion-focused coping while positively correlated with problem-focused. Perceived stress shares a negative and significant relationship with problem-focused coping and is positively correlated with emotion-focused coping. The reliability of the variables varies from 0.71 to 0.81. The skewness ranges from −0.362 to 0.14, and kurtosis varies from −0.137 to 1.533 for all variables; hence, the assumptions for normality are met (Kim, 2013).

**Figure 1:**
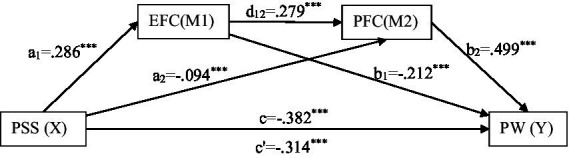
Results of serial mediation analysis. ^***^p < 0.001.

Mediation analysis is performed to examine the role of problem-focused and emotion-focused coping styles in the relationship between perceived stress and psychological well-being, as shown in Figure 1. It is clear from mediation analysis that in Table 2, that perceived stress directly predicts psychological well-being (B = −0.382, 95% CI = −0.447 to −0.316). It is also evident from Table 2 when mediators are introduced between perceived stress and psychological well-being, the coefficient value decreases (B = −0.314, 95% CI = −0.380 to −0.247). Perceived stress also predicts well-being significantly through emotion-focused coping (B = −0.061, 95% CI = -0.092 to −0.032) and problem-focused coping (B = −0.047, 95% CI = −0.075 to −0.024) as separate mediators. While it is also found that perceived stress is a predictor of psychological well-being through emotion-focused and problem-focused coping in a sequential manner (B = 0.040, 95% CI = 0.021–0.062).

**Table 2:** Descriptive statistics and correlations among variables.

	1	2	3	4
1. Perceived Stress (PSS)	1			
2. Problem-focused coping (PFC)	−0.028	1		
3. Emotion-focused coping (EFC)	0.230^**^	0.625^**^	1	
4. Psychological well-being (PW)	−0.454^**^	0.122^**^	−0.207^**^	1
Mean	17.91	16.48	49.78	48.69
SD	6.95	3.6	8.62	5.85
Skewness	0.083	−0.362	0.14	−0.211
Kurtosis	−0.045	−0.137	1.533	0.066
Alpha	0.81	0.71	0.77	0.75

** *p* < 0.01.

## Discussion

The COVID-19 pandemic needs no justification to be called a “stressful life event” for the world in many ways (health, economy, and humanitarian crisis). However, assuming the similar impact of the COVID-19 pandemic on people across the world could not be concluded as an event or a situation perceived as taxing, risky, stressful, or challenging depends on the person’s appraisal and further person-environment interactions (Lazarus and Folkman, 1984). Coping is a powerful mechanism to deal with stressors in life situations (Carver et al., 1989), becoming more important in dealing with extraordinary problems such as the COVID-19 pandemic affecting the world population with massive physical and psychological outcomes. People use different coping strategies to deal with different situations; their physical and psychological well-being depends on the selection of coping (Aldwin, 2007). Problem-focused coping is linked with higher benefits in many situations than emotion-focused (sometimes referred to as maladaptive and dysfunctional) coping styles (Boyd et al., 2009; Graven et al., 2013). However, emotional and problem-focused copings are required in many situations to deal with stressors (Folkman and Lazarus, 1985). Similarly, the present study’s findings confirm that participants use a combination of emotion-focused and problem-focused coping strategies to deal with the COVID-19 pandemic and lockdown. Past studies also confirm that psychological well-being was better for those who used both coping strategies than those who used only emotion-focused or problem-focused (Yeung and Fung, 2007; Chen, 2012; Li, 2020).

The serial mediation analysis confirmed the hypothesis that participants first chose emotion-focused coping after appraising the stressor (COVID-19 related threat and changes in regular life functioning) and then moved toward problem-focused coping, which eventually impacted their psychological well-being. Immediate emotions need to be addressed and managed to think clearly for any possible solution. Initial overwhelming emotions, stress, anxiety, and worries were the priorities for everyone to address and manage so that they could think of an action plan and arrange resources per new requirements. Past studies on life-threatening diseases, crises, and loss of loved ones suggested the effectiveness and immediate and foremost response to these situations (Carver and Scheier, 1994; Butler et al., 2005; Chen, 2012; Ben-Zur, 2020; Grover et al., 2020; Table 3).

**Table 3:** Serial mediation analysis.

Path	Effect	SE	LLCI	ULCI
PSS → EFC → PW	−0.061	0.016	−0.092	−0.032
PSS → PFC → PW	−0.047	0.013	−0.075	−0.024
PSS → EFC → PFC → PW	0.04	0.011	0.021	0.062
Total effect	−0.382	0.034	−0.447	−0.316
Direct effect	−0.314	0.034	−0.38	−0.247
Indirect effect	−0.068	0.015	−0.099	−0.04

Emotion-focused coping is generally used by individuals when they cannot actively make any difference to control the given situation; they look for alternatives to deal with it (Lazarus and Folkman, 1984). One of the emotion-focused coping mechanisms is religion which may act as a stress buffer or a way to cope during stressful events like serious illness (Carr, 2020; Siegel et al., 2001). In India, people engage in various religious rituals as part of their daily cultural practices. According to a report of Pew Research Center (2021), Indian families perform daily prayers in their homes to protect their families. Offering prayers to God and performing different rituals and practices for the welfare of the family members is encouraged in India as a part of socio-religious practices. This is supported by a 21% increase in religious activities in India during the COVID-19 pandemic compared to regular times (Fatima et al., 2022). The government also encouraged Indians to focus on religious/mythological programs, e.g., Ramayan and Mahabharat, on television as a stress management technique during COVID pandemic lockdown phases in India (Chakraborty, 2020). Another emotion-focused strategy utilized by participants was self-distraction. This strategy is commonly found at traumatic events like (e.g., 9/11) and in situations that have less control (Butler et al., 2005; Janson and Rohleder, 2017). As a part of emotion-focused coping, participants also utilized venting to cope with continued stress and emotions; however, the more they shared their negative emotions, the more stress they might experience. Venting is a two-way process with two components-one is the person who is venting, and another is the person who is hearing the vent. Due to the stressful lockdown situations, both persons were in the same condition, so it might be possible when the participant was venting her/his negative emotion to others. Individuals also receive stress-provoking information from another person during the venting process. Sharing stressful information may increase their stress because all people were experiencing the same stressful COVID-19 lockdown (Umucu and Lee, 2020; Gurvich et al., 2021).

Emotion-focused copings, such as behavioral disengagement and self-distraction, are characterized by an individual’s constantly reducing effort or completely giving up on the stressful situation by engaging in any mundane activities rather than solving the event responsible for stressful encounters. During the continued lockdown in India, participants experienced continued stress-inducing situations. However, they might not be able to find ways to make any significant contribution to changing the situations, but they could divert their attention from the current situations and future worries by engaging themselves in various non-productive activities (Greenglass et al., 2022; Gurvich et al., 2021). In the case of students, the delays in planning and executing many significant life-course events (education, job, and marriage) due to the sudden lockdown phases might evoke guilt of delaying in responding to opportunities. However, students who had the opportunity to appear for competitive exams or apply for jobs might have delayed it before the pandemic by simply thinking they were unprepared. For many others, marriage may have been delayed or canceled due to the COVID-19 pandemic, which could have been successfully completed before the pandemic arrived. Similarly, self-blame could be experienced by those individuals who had been delaying the most desired family vacations to some exotic or foreign locations, which suddenly appeared inaccessible due to the COVID-19 pandemic (Umucu and Lee, 2020; Babicka-Wirkus et al., 2021; Gurvich et al., 2021). In addition to this, positive reframing coping allows individuals to find positivity in the worst situations or even in situations with trauma. It will enable the individual to re-interpret the event positively (Mikulincer and Florian, 1996; Tennen and Affleck, 2002).

Problem-focused coping consists of active, planning, and instrumental support. Active coping refers to dealing with stressful situations by directly using available resources. The participants used these coping styles to change the nature of stressful situations or decrease stress intensity due to lockdown phases and other difficulties that arose from the pandemic. For example, during the constant lockdown phases, it was impossible for the participants to change the nature of the situation, such as uplifting the lockdown and providing a solution to the world to eliminate the COVID-19 virus. Still, they could try to reduce the perceived stress related to the COVID-19 virus and changes in life due to the lockdown by strictly following government guidelines for COVID-19 and making effective use of the time they got with their families due to lockdown phases. They planned their valuable time with their family members. During an adverse time like a lockdown and pandemic, social support from family and peers increases individuals’ psychological well-being (Wang et al., 2022).

The findings of the study are important to address similar situations like pandemics, epidemics, or crises. These situations directly impact the physical and mental health of the individuals dealing with these situations. Therefore, it is important to explore the path which connects stressors to the well-being of the individuals, and the role of coping between stressor and well-being play a significant role in determining the outcome. Lazarus and Folkman (1984) first highlighted the significance of appraisal between the stressor and its outcomes. Walinga (2008) also reintroduced the significance of appraisal through the focus of readiness and emphasized that through the change in approach it *“may be possible to facilitate a focal shift from ‘resistance’ to ‘resolution’ and from a desire for* ‘*power over’ a change to a recognition of one’s* ‘*power to’ change effectively”* (p.1, 2008).

The findings of this study could be utilized to prepare future responses by policymakers, Public health agencies, and higher government bodies (such as disaster management relief). Programs related to stress management and resilient coping could benefit individuals in pandemic situations. It will also help these health professionals to identify high-risk populations and vulnerable groups who need special attention regarding psychological issues. These findings can be extended to test the implications of the best coping strategies combinations that could lead to better physical and psychological well-being during a crisis.

Despite its prolific significance, this study had some limitations. First, convenience sampling had yielded fast responses, and data from many states of India limit the of generalization ability of the findings. Second, the sample is limited to the people who had internet access during data collection, which resulted in falling out of the population who could not be contacted without the internet.

## Data availability statement

The raw data supporting the conclusions of this article will be made available by the authors, without undue reservation.

## Ethics statement

The studies involving human participants were reviewed and approved by Research Conduct and Ethics Committee, CHRIST (Deemed to be University). The patients/participants provided their written informed consent to participate in this study.

## Author contributions

SS and PU were involved in designing the study, conceptualization, data analysis, and manuscript drafting. RJ was responsible for data collection and reviewing the final draft of the manuscript. All authors contributed to the article and approved the submitted version.

## Conflict of interest

The authors declare that the research was conducted in the absence of any commercial or financial relationships that could be construed as a potential conflict of interest.

## Publisher’s note

All claims expressed in this article are solely those of the authors and do not necessarily represent those of their affiliated organizations, or those of the publisher, the editors and the reviewers. Any product that may be evaluated in this article, or claim that may be made by its manufacturer, is not guaranteed or endorsed by the publisher.
